# Poly[μ_2_-chlorido-dichlorido[μ_2_-4′-(4-pyrid­yl)-2,2′:6′,2′′-terpyridine]­copper(I)copper(II)]

**DOI:** 10.1107/S1600536811014759

**Published:** 2011-04-29

**Authors:** Chao-Ying Zhu

**Affiliations:** a Department of Applied Chemistry, Zhejiang Sci-Tech University, Hangzhou 310018, People’s Republic of China, and Key Laboratory of Advanced Textile Materials and Manufacturing Technology, Ministry of Education, Zhejiang Sci-Tech University, Hangzhou 310018, People’s Republic of China

## Abstract

In the mixed-valence Cu^I^/Cu^II^ coordination polymer, [Cu_2_Cl_3_(C_20_H_14_N_4_)]_*n*_, the two Cu atoms are bridged to a pair of Cl atoms across a centre of inversion. The monovalent metal atoms is coordinated by a pyridine N atom as well as by three Cl atoms in a tetra­hedral CuNCl_3_ geometry. The divalent metal atom is *N*,*N*′,*N*′′-chelated by the heterocycle, and it exists in a square-pyramidal CuN_3_Cl_2_ geometry; the apical site is occupied by the second bridging Cl atom. The bridging modes of the Cl atoms and the heterocycle give rise to the formation of a layered arrangement parallel to (001).

## Related literature

For related structures, see: Hou *et al.* (2005 ▶); Zhang *et al.* (2007 ▶)
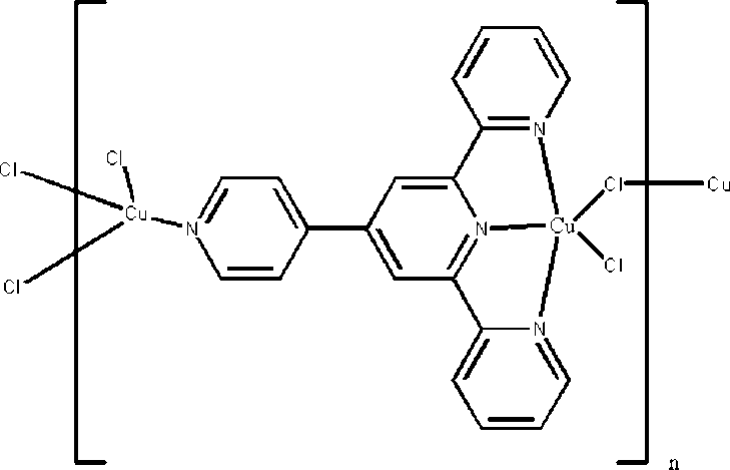


## Experimental

### 

#### Crystal data

[Cu_2_Cl_3_(C_20_H_14_N_4_)]
*M* *_r_* = 543.78
Triclinic, 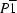

*a* = 8.1389 (8) Å
*b* = 9.8161 (10) Å
*c* = 12.4823 (13) Å
α = 79.512 (2)°
β = 85.036 (2)°
γ = 88.202 (2)°
*V* = 976.78 (17) Å^3^
*Z* = 2
Mo *K*α radiation
μ = 2.60 mm^−1^
*T* = 294 K
0.15 × 0.12 × 0.10 mm

#### Data collection

Bruker SMART diffractometer
Absorption correction: multi-scan (*SADABS*; Sheldrick, 1996 ▶) *T*_min_ = 0.694, *T*_max_ = 0.771
7840 measured reflections
3778 independent reflections
3391 reflections with *I* > 2σ(*I*)
*R*_int_ = 0.014

#### Refinement

*R*[*F* ^2^ > 2σ(*F* ^2^)] = 0.027
*wR*(*F* ^2^) = 0.072
*S* = 1.06
3778 reflections
262 parameters
H-atom parameters constrained
Δρ_max_ = 0.40 e Å^−3^
Δρ_min_ = −0.34 e Å^−3^

### 

Data collection: *SMART* (Bruker, 1998 ▶); cell refinement: *SAINT* (Bruker, 1998 ▶); data reduction: *SAINT*; program(s) used to solve structure: *SHELXS97* (Sheldrick, 2008 ▶); program(s) used to refine structure: *SHELXL97* (Sheldrick, 2008 ▶); molecular graphics: *SHELXTL* (Sheldrick, 2008 ▶); software used to prepare material for publication: *SHELXTL*.

## Supplementary Material

Crystal structure: contains datablocks I, global. DOI: 10.1107/S1600536811014759/ng5143sup1.cif

Structure factors: contains datablocks I. DOI: 10.1107/S1600536811014759/ng5143Isup2.hkl

Additional supplementary materials:  crystallographic information; 3D view; checkCIF report

## Figures and Tables

**Fig. 1.**
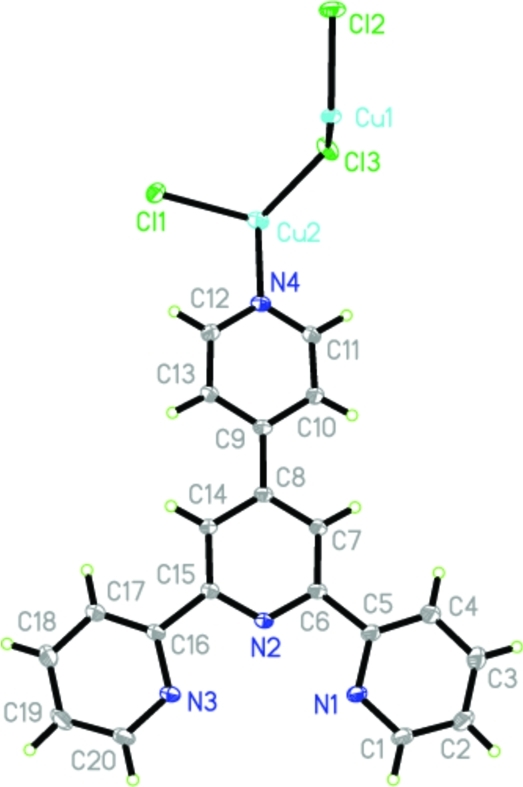
The asymmetric unit of the title compound.

**Fig. 2.**
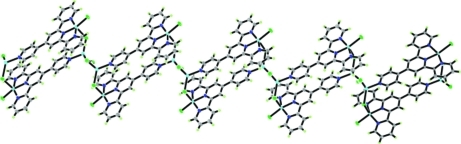
The 1-D zigzag chain structure of the title compoud.

## supplementary crystallographic information

### Comment

Terpyridine and its derivatives have been receiving rapidly increasing attention recently not only because of their versatility as building blocks in supramolecular assembles, but also due to the interesting electronic, photonic and magnetic properties of their transition metal complexes.

4'-(4-Pyridyl)-2,2':6'2''-terpyridine(pyterpy) belongs to this group of ligands and has usually been used to construct a great variety of structurally interesting entities, such as ribbon-type coordination polymers (Hou *et al.*, 2005) and self-catenated networks (Zhang *et al.*, 2007).

The structure of the title compound (I) is shown in Fig. 1. Single-crystal X-ray diffraction shows that the asymmetric unit contains two Cu crystallographically nonequivalent atoms. The Cu1 atom has a distorted square-pyramidal coordination formed by three N atoms of tridentate 4'-(4-pyridyl)-2,2':6'2''-terpyridine (pyterpy) ligand and two Cl atoms. The Cu2 atom is coordinated by one N atom from the pendent monodentate pyridine of pyterpy as well as by three Cl atoms, conferring a tetrahedral coordination geometry. The two terpy ligands in a *transoid* arrangement link Cu1 and Cu2 atoms, to form a mixed-valence tetrameric *M*_4_*L*_4_ rectangular unit with a separation of 11.017 Å, which is smaller than those in reported ribbon-type compounds, and then linked by a Cu_2_Cl_2_ cluster, leading to the formation of an infinite 1-D coordination polymer (Fig. 2).

### Experimental

The mixture of CuCl (0.020 g, 0.2 mmol), 4'-(4-pyridyl)-2,2':6'2''-terpyridine (pyterpy) (0.062 g, 0.1 mmol), and acetonitrile (6 ml) were placed and sealed in a 15 ml Teflon-lined stainless steel reactor and heated to 180 °C for 72 h, then cooled down to room temperature at a rate of 2 °C/ 20 min. Single crystals suitable for X-ray diffraction were obtained in the form of black bars in *ca* 20% yield.

### Refinement

H atoms were positioned geometrically and refined using a riding model, with C—H = 0.93 Å (aromatic) and *U*_iso_(H) = 1.2*U*_eq_(C)

### Crystal data

**Table:**

[Cu_2_Cl_3_(C_20_H_14_N_4_)]	*Z* = 2
*M**_r_* = 543.78	*F*(000) = 542
Triclinic, *P*1	*D*_x_ = 1.849 Mg m^−^^3^
Hall symbol: -P 1	Mo *K*α radiation, λ = 0.71073 Å
*a* = 8.1389 (8) Å	Cell parameters from 1056 reflections
*b* = 9.8161 (10) Å	θ = 2.4–26.0°
*c* = 12.4823 (13) Å	µ = 2.60 mm^−^^1^
α = 79.512 (2)°	*T* = 294 K
β = 85.036 (2)°	Block, black
γ = 88.202 (2)°	0.15 × 0.12 × 0.10 mm
*V* = 976.78 (17) Å^3^	

### Data collection

**Table:**

Bruker SMART diffractometer	3778 independent reflections
Radiation source: fine-focus sealed tube	3391 reflections with *I* > 2σ(*I*)
graphite	*R*_int_ = 0.014
φ and ω scans	θ_max_ = 26.0°, θ_min_ = 2.1°
Absorption correction: multi-scan (*SADABS*; Sheldrick, 1996)	*h* = −10→9
*T*_min_ = 0.694, *T*_max_ = 0.771	*k* = −12→12
7840 measured reflections	*l* = −15→15

### Refinement

**Table:**

Refinement on *F*^2^	Primary atom site location: structure-invariant direct methods
Least-squares matrix: full	Secondary atom site location: difference Fourier map
*R*[*F*^2^ > 2σ(*F*^2^)] = 0.027	Hydrogen site location: inferred from neighbouring sites
*wR*(*F*^2^) = 0.072	H-atom parameters constrained
*S* = 1.06	*w* = 1/[σ^2^(*F*_o_^2^) + (0.0383*P*)^2^ + 0.3983*P*] where *P* = (*F*_o_^2^ + 2*F*_c_^2^)/3
3778 reflections	(Δ/σ)_max_ = 0.002
262 parameters	Δρ_max_ = 0.40 e Å^−^^3^
0 restraints	Δρ_min_ = −0.34 e Å^−^^3^

### Special details

**Table:**

Geometry. All e.s.d.'s (except the e.s.d. in the dihedral angle between two l.s. planes) are estimated using the full covariance matrix. The cell e.s.d.'s are taken into account individually in the estimation of e.s.d.'s in distances, angles and torsion angles; correlations between e.s.d.'s in cell parameters are only used when they are defined by crystal symmetry. An approximate (isotropic) treatment of cell e.s.d.'s is used for estimating e.s.d.'s involving l.s. planes.
Refinement. Refinement of *F*^2^ against ALL reflections. The weighted *R*-factor *wR* and goodness of fit *S* are based on *F*^2^, conventional *R*-factors *R* are based on *F*, with *F* set to zero for negative *F*^2^. The threshold expression of *F*^2^ > σ(*F*^2^) is used only for calculating *R*-factors(gt) *etc*. and is not relevant to the choice of reflections for refinement. *R*-factors based on *F*^2^ are statistically about twice as large as those based on *F*, and *R*-factors based on ALL data will be even larger.

### Fractional atomic coordinates and isotropic or equivalent isotropic displacement parameters (Å^2^)

**Table:**

	*x*	*y*	*z*	*U*_iso_*/*U*_eq_	
Cu1	1.55686 (3)	0.36696 (3)	−0.35707 (2)	0.02849 (9)	
Cu2	1.49349 (3)	0.14312 (3)	−0.05428 (3)	0.04087 (10)	
Cl1	1.36557 (7)	−0.05058 (6)	−0.10452 (5)	0.03600 (14)	
Cl3	1.68201 (7)	0.25884 (6)	−0.18347 (5)	0.03968 (15)	
Cl2	1.73952 (7)	0.30031 (7)	−0.48213 (5)	0.04485 (16)	
C16	0.4539 (2)	0.3584 (2)	0.30399 (17)	0.0271 (4)	
N2	0.6324 (2)	0.54582 (18)	0.28869 (14)	0.0266 (4)	
N4	1.2937 (2)	0.24557 (19)	0.00438 (16)	0.0332 (4)	
N3	0.3521 (2)	0.43980 (19)	0.35802 (14)	0.0299 (4)	
N1	0.6210 (2)	0.77686 (18)	0.35354 (14)	0.0288 (4)	
C11	1.3087 (3)	0.3651 (2)	0.03817 (19)	0.0333 (5)	
H11	1.4103	0.4084	0.0239	0.040*	
C15	0.6150 (2)	0.4212 (2)	0.26284 (17)	0.0265 (4)	
C14	0.7417 (2)	0.3610 (2)	0.20518 (17)	0.0282 (4)	
H14	0.7274	0.2755	0.1854	0.034*	
C9	1.0297 (2)	0.3681 (2)	0.11560 (17)	0.0273 (4)	
C8	0.8918 (2)	0.4302 (2)	0.17696 (17)	0.0260 (4)	
C6	0.7742 (2)	0.6135 (2)	0.26509 (17)	0.0270 (4)	
C7	0.9077 (2)	0.5576 (2)	0.20962 (17)	0.0286 (4)	
H7	1.0068	0.6047	0.1944	0.034*	
C10	1.1832 (3)	0.4291 (2)	0.09312 (19)	0.0324 (5)	
H10	1.2014	0.5126	0.1150	0.039*	
C1	0.6025 (3)	0.8956 (2)	0.39099 (19)	0.0355 (5)	
H1	0.5002	0.9170	0.4237	0.043*	
C3	0.8787 (3)	0.9570 (3)	0.3336 (2)	0.0464 (6)	
H3	0.9655	1.0182	0.3272	0.056*	
C18	0.2576 (3)	0.1787 (3)	0.3369 (2)	0.0415 (6)	
H18	0.2260	0.0904	0.3308	0.050*	
C4	0.9006 (3)	0.8347 (2)	0.2933 (2)	0.0404 (6)	
H4	1.0018	0.8122	0.2598	0.049*	
C5	0.7688 (3)	0.7472 (2)	0.30392 (17)	0.0286 (4)	
C17	0.4102 (3)	0.2284 (2)	0.29147 (19)	0.0335 (5)	
H17	0.4816	0.1749	0.2532	0.040*	
C2	0.7283 (3)	0.9875 (3)	0.3832 (2)	0.0411 (6)	
H2	0.7120	1.0691	0.4109	0.049*	
C13	1.0125 (3)	0.2455 (3)	0.0776 (2)	0.0465 (7)	
H13	0.9113	0.2014	0.0886	0.056*	
C20	0.2040 (3)	0.3911 (3)	0.40001 (19)	0.0368 (5)	
H20	0.1330	0.4468	0.4364	0.044*	
C19	0.1529 (3)	0.2610 (3)	0.3913 (2)	0.0420 (6)	
H19	0.0496	0.2296	0.4216	0.050*	
C12	1.1452 (3)	0.1887 (3)	0.0232 (2)	0.0480 (7)	
H12	1.1300	0.1065	−0.0014	0.058*	

### Atomic displacement parameters (Å^2^)

**Table:**

	*U*^11^	*U*^22^	*U*^33^	*U*^12^	*U*^13^	*U*^23^
Cu1	0.02035 (14)	0.03193 (16)	0.03487 (16)	0.00211 (10)	0.00583 (10)	−0.01466 (11)
Cu2	0.02869 (16)	0.04265 (18)	0.0509 (2)	0.00035 (12)	0.01106 (13)	−0.01440 (14)
Cl1	0.0362 (3)	0.0355 (3)	0.0399 (3)	−0.0021 (2)	−0.0025 (2)	−0.0165 (2)
Cl3	0.0261 (3)	0.0494 (3)	0.0401 (3)	−0.0083 (2)	0.0011 (2)	0.0003 (3)
Cl2	0.0350 (3)	0.0568 (4)	0.0461 (3)	0.0020 (3)	0.0134 (2)	−0.0265 (3)
C16	0.0199 (10)	0.0300 (11)	0.0308 (11)	0.0016 (8)	0.0031 (8)	−0.0069 (9)
N2	0.0191 (8)	0.0270 (9)	0.0340 (9)	0.0018 (7)	0.0035 (7)	−0.0092 (7)
N4	0.0229 (9)	0.0345 (10)	0.0428 (11)	0.0016 (7)	0.0081 (8)	−0.0140 (8)
N3	0.0234 (9)	0.0323 (10)	0.0334 (10)	0.0012 (7)	0.0061 (7)	−0.0085 (8)
N1	0.0244 (9)	0.0311 (9)	0.0335 (9)	0.0033 (7)	−0.0003 (7)	−0.0143 (8)
C11	0.0208 (10)	0.0339 (12)	0.0455 (13)	−0.0020 (9)	0.0067 (9)	−0.0120 (10)
C15	0.0211 (10)	0.0263 (10)	0.0319 (11)	0.0001 (8)	0.0028 (8)	−0.0073 (8)
C14	0.0228 (10)	0.0270 (10)	0.0359 (11)	−0.0002 (8)	0.0051 (8)	−0.0121 (9)
C9	0.0217 (10)	0.0289 (11)	0.0315 (11)	0.0030 (8)	0.0033 (8)	−0.0092 (9)
C8	0.0211 (10)	0.0285 (11)	0.0291 (10)	0.0023 (8)	0.0016 (8)	−0.0094 (8)
C6	0.0203 (10)	0.0284 (11)	0.0331 (11)	0.0024 (8)	0.0015 (8)	−0.0099 (9)
C7	0.0199 (10)	0.0309 (11)	0.0365 (12)	−0.0011 (8)	0.0035 (8)	−0.0125 (9)
C10	0.0235 (11)	0.0306 (11)	0.0452 (13)	−0.0013 (9)	0.0034 (9)	−0.0149 (10)
C1	0.0316 (12)	0.0363 (12)	0.0416 (13)	0.0062 (10)	0.0012 (10)	−0.0184 (10)
C3	0.0365 (13)	0.0401 (14)	0.0682 (18)	−0.0090 (11)	−0.0012 (12)	−0.0244 (13)
C18	0.0294 (12)	0.0366 (13)	0.0583 (16)	−0.0068 (10)	0.0024 (11)	−0.0096 (11)
C4	0.0261 (11)	0.0393 (13)	0.0595 (15)	−0.0017 (10)	0.0031 (10)	−0.0213 (12)
C5	0.0238 (10)	0.0294 (11)	0.0342 (11)	0.0038 (8)	−0.0005 (8)	−0.0117 (9)
C17	0.0232 (11)	0.0334 (12)	0.0444 (13)	−0.0002 (9)	0.0043 (9)	−0.0115 (10)
C2	0.0433 (14)	0.0348 (13)	0.0504 (14)	0.0015 (10)	−0.0019 (11)	−0.0231 (11)
C13	0.0246 (11)	0.0452 (14)	0.0749 (18)	−0.0108 (10)	0.0186 (11)	−0.0334 (13)
C20	0.0240 (11)	0.0434 (13)	0.0411 (13)	0.0019 (10)	0.0092 (9)	−0.0087 (10)
C19	0.0225 (11)	0.0462 (14)	0.0538 (15)	−0.0075 (10)	0.0082 (10)	−0.0044 (12)
C12	0.0326 (13)	0.0413 (14)	0.0762 (19)	−0.0075 (11)	0.0179 (12)	−0.0361 (13)

### Geometric parameters (Å, °)

**Table:**

Cu1—N2^i^	1.9466 (16)	C14—H14	0.9300
Cu1—N1^i^	2.0455 (18)	C9—C10	1.387 (3)
Cu1—N3^i^	2.0557 (18)	C9—C13	1.387 (3)
Cu1—Cl2	2.2325 (6)	C9—C8	1.483 (3)
Cu1—Cl3	2.5172 (6)	C8—C7	1.397 (3)
Cu2—N4	2.0374 (18)	C6—C7	1.390 (3)
Cu2—Cl3	2.2933 (6)	C6—C5	1.478 (3)
Cu2—Cl1^ii^	2.3964 (7)	C7—H7	0.9300
Cu2—Cl1	2.4007 (6)	C10—H10	0.9300
Cu2—Cu2^ii^	2.8917 (7)	C1—C2	1.371 (3)
Cl1—Cu2^ii^	2.3964 (7)	C1—H1	0.9300
C16—N3	1.355 (3)	C3—C2	1.373 (3)
C16—C17	1.375 (3)	C3—C4	1.385 (3)
C16—C15	1.477 (3)	C3—H3	0.9300
N2—C6	1.333 (3)	C18—C19	1.377 (3)
N2—C15	1.335 (3)	C18—C17	1.385 (3)
N2—Cu1^i^	1.9466 (16)	C18—H18	0.9300
N4—C11	1.330 (3)	C4—C5	1.377 (3)
N4—C12	1.332 (3)	C4—H4	0.9300
N3—C20	1.339 (3)	C17—H17	0.9300
N3—Cu1^i^	2.0557 (18)	C2—H2	0.9300
N1—C1	1.332 (3)	C13—C12	1.382 (3)
N1—C5	1.353 (3)	C13—H13	0.9300
N1—Cu1^i^	2.0455 (18)	C20—C19	1.381 (3)
C11—C10	1.380 (3)	C20—H20	0.9300
C11—H11	0.9300	C19—H19	0.9300
C15—C14	1.385 (3)	C12—H12	0.9300
C14—C8	1.403 (3)		
			
N2^i^—Cu1—N1^i^	78.97 (7)	C10—C9—C8	121.92 (19)
N2^i^—Cu1—N3^i^	79.19 (7)	C13—C9—C8	121.8 (2)
N1^i^—Cu1—N3^i^	156.19 (7)	C7—C8—C14	118.17 (18)
N2^i^—Cu1—Cl2	162.20 (6)	C7—C8—C9	121.33 (19)
N1^i^—Cu1—Cl2	99.27 (5)	C14—C8—C9	120.49 (19)
N3^i^—Cu1—Cl2	98.61 (5)	N2—C6—C7	120.70 (19)
N2^i^—Cu1—Cl3	96.91 (5)	N2—C6—C5	112.78 (17)
N1^i^—Cu1—Cl3	97.93 (5)	C7—C6—C5	126.51 (19)
N3^i^—Cu1—Cl3	93.99 (5)	C6—C7—C8	119.42 (19)
Cl2—Cu1—Cl3	100.87 (2)	C6—C7—H7	120.3
N4—Cu2—Cl3	120.37 (6)	C8—C7—H7	120.3
N4—Cu2—Cl1^ii^	104.34 (6)	C11—C10—C9	119.6 (2)
Cl3—Cu2—Cl1^ii^	107.93 (2)	C11—C10—H10	120.2
N4—Cu2—Cl1	101.32 (6)	C9—C10—H10	120.2
Cl3—Cu2—Cl1	115.66 (2)	N1—C1—C2	122.6 (2)
Cl1^ii^—Cu2—Cl1	105.86 (2)	N1—C1—H1	118.7
N4—Cu2—Cu2^ii^	111.61 (6)	C2—C1—H1	118.7
Cl3—Cu2—Cu2^ii^	127.92 (2)	C2—C3—C4	119.6 (2)
Cl1^ii^—Cu2—Cu2^ii^	52.998 (16)	C2—C3—H3	120.2
Cl1—Cu2—Cu2^ii^	52.861 (18)	C4—C3—H3	120.2
Cu2^ii^—Cl1—Cu2	74.14 (2)	C19—C18—C17	119.5 (2)
Cu2—Cl3—Cu1	112.90 (2)	C19—C18—H18	120.2
N3—C16—C17	122.26 (19)	C17—C18—H18	120.2
N3—C16—C15	113.85 (18)	C5—C4—C3	118.4 (2)
C17—C16—C15	123.88 (18)	C5—C4—H4	120.8
C6—N2—C15	121.47 (17)	C3—C4—H4	120.8
C6—N2—Cu1^i^	119.54 (14)	N1—C5—C4	121.9 (2)
C15—N2—Cu1^i^	118.95 (14)	N1—C5—C6	113.76 (18)
C11—N4—C12	116.29 (19)	C4—C5—C6	124.37 (19)
C11—N4—Cu2	121.51 (15)	C16—C17—C18	118.6 (2)
C12—N4—Cu2	121.71 (15)	C16—C17—H17	120.7
C20—N3—C16	118.36 (19)	C18—C17—H17	120.7
C20—N3—Cu1^i^	127.42 (15)	C1—C2—C3	118.8 (2)
C16—N3—Cu1^i^	114.11 (14)	C1—C2—H2	120.6
C1—N1—C5	118.65 (19)	C3—C2—H2	120.6
C1—N1—Cu1^i^	126.56 (15)	C12—C13—C9	120.2 (2)
C5—N1—Cu1^i^	114.75 (14)	C12—C13—H13	119.9
N4—C11—C10	124.2 (2)	C9—C13—H13	119.9
N4—C11—H11	117.9	N3—C20—C19	122.4 (2)
C10—C11—H11	117.9	N3—C20—H20	118.8
N2—C15—C14	120.84 (19)	C19—C20—H20	118.8
N2—C15—C16	113.24 (17)	C18—C19—C20	118.9 (2)
C14—C15—C16	125.90 (19)	C18—C19—H19	120.6
C15—C14—C8	119.30 (19)	C20—C19—H19	120.6
C15—C14—H14	120.4	N4—C12—C13	123.4 (2)
C8—C14—H14	120.4	N4—C12—H12	118.3
C10—C9—C13	116.26 (19)	C13—C12—H12	118.3

Symmetry codes: (i) −*x*+2, −*y*+1, −*z*; (ii) −*x*+3, −*y*, −*z*.
